# The prevalence of COVID-19 infection, associated risk factors and post-COVID-19 symptoms among vaccinated people, in Anhui Province, China: A cross-sectional study

**DOI:** 10.1097/MD.0000000000037366

**Published:** 2024-03-08

**Authors:** Tianyun Yu, Yujia Zhai, Can Cui, Zengfeng Su

**Affiliations:** a Department of General Medicine, Chaohu Hospital affiliated with Anhui Medical University, Chaohu, China.

**Keywords:** COVID-19 infection, COVID-19 symptoms, long COVID19, post-COVID-19 syndrome, postvaccination COVID-19, risk factors

## Abstract

To investigate the infection status of coronavirus disease 2019 (COVID-19) among people in Anhui Province, China after the epidemic prevention and control measures were lifted, and to study and analyze its related influencing factors. From March 11 to May 20, 2023, questionnaires on COVID-19 were distributed on the Questionnaire Star platform, and Statistical Product and Service Solutions software (version 19.0) was used for statistical processing. The results showed that the infection rate of COVID-19 among respondents reached 72.24%. 58.81% of the infected people reported post COVID-19 symptoms. Fever, fatigue, and cough were the main symptoms during infection. The results of multi-factor logistic regression analysis showed that there is statistical significance between age (*P* = .002), residential area (*P* = .025), number of vaccine injections (*P* < .001) and the risk of new coronavirus infection. COVID-19 had a high infection rate, and children had a lower risk of COVID-19. People living in cities were more susceptible to COVID-19, and it was necessary to increase the number of vaccine doses.

**Key points**

- The global infection rate of the new coronavirus is steadily increasing, leading to significant effects on both life and health.
- Continuous mutation of the virus strains significantly diminished the protective effect of vaccination.
- Additionally, the overlapping symptoms of long-term new coronavirus infections and underlying diseases pose challenges in the diagnosis and treatment of the disease.
- The new coronavirus infection has a high transmission rate within the population, with over half of infected individuals reporting postinfection symptoms.
- Children have a lower risk of contracting the new coronavirus compared than adults do.
- Individuals residing in urban areas were more vulnerable to COVID-19.
- Increasing the number of vaccinations is essential for vaccinated groups as it reduces the risk of COVID-19 infection.
- In future, it is necessary to establish dedicated outpatient clinics for COVID-19 symptoms. These clinics should focus not only on effectively treating patients’ physical symptoms, but also on addressing their psychological needs. Given that the new coronavirus infection is an ongoing battle, the significance of vaccination cannot be overlooked. It is crucial to implement preventive measures and provide education specifically for patients with underlying diseases.

## 1. Introduction

Since the outbreak of the coronavirus disease 2019 (COVID-19) epidemic, China’s epidemic prevention and control policy has been strict. On December 7, 2022, the Joint Prevention and Control Mechanism of the State Council of China issued 10 new articles. Since then, preventive measures have gradually been lifted. The infection rate of COVID-19 in the population has shown a sharp upward trend. Many symptoms appear during infection, and long-term symptoms may be difficult to resolve. Studies have shown that risk factors for severe COVID-19 include older age, male sex, hypertension, diabetes, chronic obstructive pulmonary disease, and cardiovascular disease,^[1]^ whether these factors also affect the risk of COVID-19 infection remains uncertain. COVID-19 has had a serious impact on the health of the population. To understand the current infection status and epidemic trend of COVID-19 in the population and to explore the influencing factors related to the occurrence of COVID-19, this survey collected relevant data and conducted statistical analysis to provide scientific basis for preventing COVID-19 infection.

## 2. Materials and methods

### 2.1. Research object

From March 11 to May 20, 2023, Questionnaire Star was used to publish a self-compiled questionnaire to investigate the COVID-19 infection situation among people in Anhui Province, China after the epidemic prevention and control measures were relaxed. A total of 1135 people completed the questionnaires; 184 people were excluded because of incomplete information, and 951 were ultimately included in this study. All survey subjects signed the informed consent was obtained from all subjects and their legal guardian(s), and this project was approved by the Medical Ethics Committee of Chaohu Hospital affiliated with Anhui Medical University.

### 2.2. Epidemiological investigation

An online questionnaire survey was adopted that included demographic information, blood type, number of vaccine doses, underlying disease history, smoking status, area of residence, and symptoms experienced during the infection. Using a list of 21 symptoms, respondents were asked to list their symptoms. Symptoms included fever, fatigue, dizziness, muscle aches, cough, and sore throat. These symptoms were selected based on a literature search and clinician’s experience. Age was grouped according to the latest classification standard. A positive COVID-19 nucleic acid test result was the primary criterion for diagnosis, followed by a positive test result for the COVID-19 antigen. According to the standards of the World Health Organization, a person with continuous or cumulative smoking for 6 months or more in a lifetime is defined as a smoker. People with post-COVID-19 symptoms were defined as having persistent symptoms after 4 weeks of COVID-19 infection.

### 2.3. Statistical methods

After the questionnaire was returned, 2 individuals entered the data simultaneously. Statistical Product and Service Solutions 19.0 software was used for statistical analysis. Univariate analysis selected gender, age, blood type, number of vaccination doses, smoking status, regular exercise, hypertension, and diabetes as independent variables, and COVID-19 infection as the outcome variable. The measurement data are expressed as the mean ± standard deviation (*x̅* ±s). The comparison of data between the groups was performed using *t*-tests. Count data are expressed as frequencies and percentiles. Comparisons between the groups were performed using the χ^2^ test. Odds ratios and 95% confidence intervals of the relevant influencing factors were analyzed using an unconditional logistic regression model. Using a two-sided statistical test, statistical significance was set at *P* < .05.

## 3. Results

### 3.1. Current status of COVID-19 infections in the population

**Figure 1.**
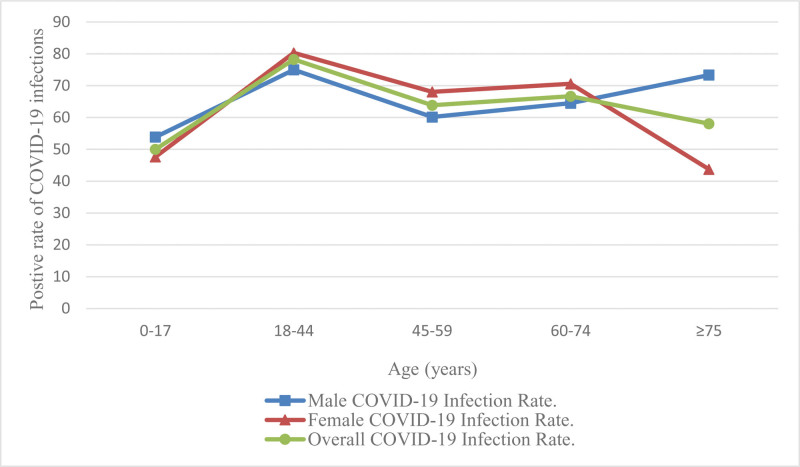
Comparison of positive rates of new coronavirus infection in different age groups. The abscissa represents age (years), and the ordinate represents the positivity rate of COVID-19 infection (%). As can be seen from the figure, the female COVID-19 infection rate is the highest among the 3 age groups of 18 to 44 years old, 45 to 59 years old, and 60 to 74 years old; the COVID-19 infection rate among men in the 2 age groups of 0 to 17 years old and 75 years old and above lowest.

The positive rate for COVID-19 infection was 72.24%. The positivity rate among women was 74.95%, which was higher than that among men (68.81%). However, no significant difference in COVID-19 infection rate was observed between the sexes. Further details are provided in Table 1. In addition, the infection rate in the young population showed an upward trend, whereas the infection rate among female children and those over 75 years of age showed a downward trend, and the overall infection rate change curve showed an “M” shape. The COVID-19 infection rate in each age group is shown in Figure 1.

**Table 1:** Comparison of positive rates of COVID-19 infection in different age groups [people (%)].

Age group (years)	Number of people	Males	Females
0–17	17 (50.00)	7 (53.85)	10 (47.62)
18–44	461 (78.27)	171 (75.00)	290 (80.33)
45–59	159 (63.86)	80 (60.15)	79 (68.10)
60–74	32 (66.67)	20 (64.52)	12 (70.59)
≥75	18 (58.06)	11 (73.33)	7 (43.75)
Total	687 (72.24)	289 (68.81)	398 (74.95)

### 3.2. Symptoms and treatment of COVID-19 infections

We performed descriptive analysis on the data and found that cough (85.3%), fatigue (84.67%), fever (83.99%), expectoration (71.32%), dizziness and headache (69.14%), muscle aches (68.85%), and sore throat (62.15%) were the main symptoms that occurred during COVID-19 infection. Among the treatments, home treatment accounted for 81.37%, outpatient treatment accounted for 5.97%, hospitalization accounted for 1.46%, and 11.21% of infected people did not take any treatment measures.

### 3.3. Post-COVID-19 symptoms

**Figure 2.**
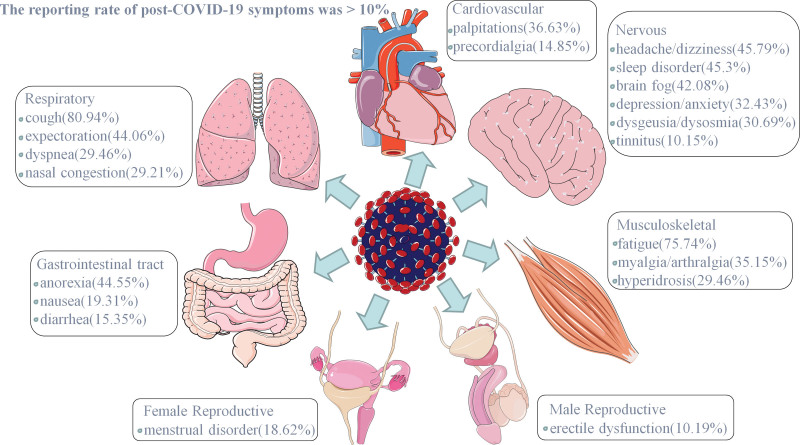
The reporting rate of post-COVID-19 symptoms was >10%. As can be seen from the figure, the respiratory and musculoskeletal systems have the highest incidence of post-COVID-19 symptoms, with cough and fatigue accounting for the highest proportions. The nervous system has the most symptom categories, with dizziness, insomnia, and brain fog occupying the top 3 categories. Fortunately, the impact of COVID-19 symptoms on the human reproductive system is low.

Among the individuals who reported post-COVID-19 symptoms, the number of people who reported respiratory and neuromotor system symptoms was the largest, and the number of people who reported reproductive system symptoms was the smallest. Among respiratory system symptoms, cough had the highest incidence (80.94%); among nervous system symptoms, headache and dizziness had the highest incidence (45.79%); and among cardiovascular system symptoms, palpitations had the highest incidence (36.63%). Among the symptoms of the skeletal system, the incidence of fatigue was the highest (75.74%); among the symptoms of the digestive system, the incidence of anorexia was the highest (44.55%); among the symptoms of the reproductive system, the incidence of menstrual cycle disorders in women (18.62%), and erectile dysfunction in men (10.19%). The reporting rate of post-COVID-19 symptoms was >10% (Fig. 2).

### 3.4. Factors significantly associated with COVID-19 infections

After reviewing a large amount of literature, we selected gender, age, blood type, number of vaccinations, area of residence, smoking, exercise, hypertension, and diabetes as independent variables, and COVID-19 infection as the outcome variable, excluding digestive system diseases, chronic kidney disease, and Immune system diseases and other factors that are not significantly related to COVID-19 infection. The variables were screened with *P* < .05, and the results showed that sex (*P* = .036), age (*P* < .001), vaccine dose (*P* = .011), area of residence (*P* < .001), history of cerebral infarction or cerebral hemorrhage (*P* = .016), and smoking (*P* = .003) were statistically significant. The univariate analysis of the statistical data is presented in Table 2.

**Table 2:** Univariate analysis of influencing factors related to COVID-19.

Variables	Number of people	Infected	Uninfected	χ^2^ value	*P* value	OR (95% CI)
Sex Female Male	531 420	398 289	133 131	4.413	0.036	1.36 (1.02–1.80) 1.00
Age (years) ≤17 18–44 45–59 60–74 ≥75	34 589 249 48 31	17 461 159 32 18	17 128 90 16 13	31.683	0.000 0.515 0.011 0.529 0.439	0.72 (0.27–1.93) 2.60 (1.24–5.45) 1.28 (0.60–2.73) 1.44 (0.57–3.67) 1.00
Blood type Type A Type B Type AB Type O	211 242 78 256	160 180 62 188	51 62 16 68	1.296	0.731	1.14 (0.75–1.73) 1.05 (0.70–1.57) 1.40 (0.76–2.59) 1.00
Number of vaccine doses 0 1 2 3 4	30 16 135 734 36	13 12 99 540 23	17 4 36 194 13	14.540	0.011 0.097 0.433 0.268 0.204	0.43 (0.16–1.67) 1.70 (0.45–6.35) 1.55 (0.71–3.39) 1.57 (0.78–3.17) 1.00
Area of residence Urban area Rural area	730 221	564 123	166 98	39.483	0.000	2.71 (1.97–3.72) 1.00
Smoking Yes No	132 819	81 606	51 213	9.041	0.003	0.56 (0.38–0.82) 1.00
Regular exercise Yes No	320 631	234 453	84 178	0.188	0.664	1.07 (0.79–1.45) 1.00
Hypertension Yes No	126 825	87 600	39 225	0.738	0.391	0.84 (0.56–1.26) 1.00
Diabetes Yes No	27 924	20 667	7 257	0.047	0.829	1.10 (0.46–2.64) 1.00

CI = confidence intervals, OR = odds ratio.

## 4. Discussion

The COVID-19 infection rate has continued to rise owing to the lifting of epidemic measures in China. Within 3 weeks after the restrictions were lifted, a study showed that 70% of the population in Macau was infected with COVID-19.^[2]^ Many people still experience a series of symptoms after contracting COVID-19, but there is no precise definition for these long-lasting symptoms. The WHO defines those with post-COVID-19 symptoms as individuals with possible or confirmed COVID-19 who still have symptoms 3 months after infection, symptoms that persist for at least 2 months, and symptoms that cannot be explained by other diseases.^[3]^ The National Institute for Health and Care Excellence defines it as symptoms that persist for more than 12 weeks after being infected with COVID-19.^[4]^ The definition adopted by the Centers for Disease Control and Prevention is persistent symptoms or health problems 4 weeks after infection with COVID-19^[5]^; this study used the definition criteria of the Centers for Disease Control and Prevention. We observed a wide range of long-term symptoms involving major systems of the body, which indicates that in addition to direct infection, long-term symptoms may also be caused by endothelial damage, immune system disorders, and hypercoagulable states.^[6]^ The quantification of viral ribonucleic acid is at a persistently low level,^[7]^ and this potentially persistent infection may explain the occurrence of symptoms after COVID-19 infection.

In this population-based survey study, we assessed various potential risk factors for COVID-19 and found that age, area of residence, and number of vaccine doses were associated with infection risk. For the other factors investigated, including sex, blood type, smoking, and other chronic diseases, no statistically significant associations with infection risk were observed. An unconditional logistic regression model showed that age (*P* = .002), area of residence (*P* < .001), and vaccine doses (*P* = .025) were statistically significant, the infection risk was the lowest in the ≤17 age group and the highest in the 18 to 44 age the risk of infection was higher in urban areas. The risk of infection with one dose of vaccine was the highest. A large epidemiological study in China observed that the age distribution of COVID-19 cases was skewed toward an older population, with a median age of 45 years and lower infection rate among children. Among people over 19 years of age, the likelihood of COVID-19 increased sharply.^[8]^ A study in the UK also showed that children and adolescents had a very low risk of developing COVID-19, and the vast majority of confirmed infected children and adolescents had only mild or even no symptoms,^[9]^ which is consistent with our findings. Compared with young people, some people who are older or have underlying diseases may have a reduced risk of infection by reducing social activities and the number of times they enter and exit public places. A large proportion of the population remains unvaccinated,^[10]^ which may be the reason for the reduced prevalence and infection risk in the unvaccinated population. The risk of infection in urban areas may be significantly higher than in rural areas because of the dense population and frequent social contact. In our study, there was no significant difference in the susceptibility of people of either sex to COVID-19, but some studies have suggested that the severity and mortality of COVID-19 among men are higher than those among women.^[11]^ Many studies have shown that the A blood type is associated with an increased risk of infection, while the O blood type is associated with a reduced risk.^[12]^ Our study failed to reach this result, and some studies have found that the ABO blood type is not associated with susceptibility.^[13]^ Although studies have shown that nicotine upregulates the expression of angiotensin-converting enzyme 2 and increases susceptibility to COVID-19,^[14]^ we included smoking in the multivariate regression model analysis, and the results were not statistically significant. This study also showed that older age, male sex, and underlying diseases such as hypertension, coronary heart disease, and chronic obstructive pulmonary disease did not increase the risk of COVID-19, and there was no association between chronic kidney disease and susceptibility, which is consistent with the findings of de Lusignan et al.^[15]^ However, the findings of this study are inconsistent. This discrepancy may be related to the fact that the questionnaire used affected the reliability of the results, or it may have been a false-positive result caused by insufficient adjustment for potential confounding factors in other studies. Regarding research on post-COVID-19 symptoms, A 2019 Dutch observational cohort study on symptoms after COVID-19 infection found that symptoms after COVID-19 infection included chest pain, difficulties with breathing, pain when breathing, painful muscles, ageusia or anosmia, tingling extremities, lump in throat, feeling hot and cold alternately, heavy arms or legs, and general tiredness.^[16]^ This is consistent with the results of this study. And in 12.7% of patients, these symptoms were attributable to COVID-19 infection. Also a French study on self-reported COVID-19 infection and SARS-CoV-2 serological testing of adults with persistent physical symptoms during the COVID-19 pandemic showed that among 26,823 volunteers participating in the study, self-reported infection was positively associated with persistent physical symptoms, whereas a positive SARS-COV-2 serology test result was only positively associated with persistent olfactory impairment.^[17]^

## 5. Limitations

Since the scope of the survey focused on COVID-19 infections after the lifting of epidemic prevention and control measures in the Anhui Province, there were certain limitations, and there was no comparison with the infection situation in other regions. Additionally, the content of the questionnaire was written by one of the authors. The questionnaire included multiple-choice questions and fill-in-the-blank questions; thus, it was difficult to ensure the reliability and validity of the questionnaire. Due to the age structure of Internet users, there may have been some imbalance in the age distribution of respondents. The questionnaire format made it difficult to rule out the possibility of false positives or false negatives in the sample, and asymptomatic infections could not be identified. Owing to the controversial diagnostic criteria for post-COVID-19 symptoms, there is currently no uniform biomarker or imaging test that can be used to confirm the diagnosis. Symptoms such as mild fatigue and decreased concentration are common in the general population and cannot be clinically ruled out. Psychological or other factors interfered, and the descriptions of symptoms after COVID-19 infection in the survey were all from the self-reports of the respondents. In short, this study evaluated the current situation, and the relationship between the research factors and the conclusions obtained was exploratory. Further observations and more in-depth research are required to clarify this correlation.

## Acknowledgments

We would like to thank Zengfeng Su team in general practice at the Chaohu Hospital of Anhui Medical University for their valuable help.

## Author contributions

**Formal analysis:** Can Cui.

**Investigation:** Yujia Zhai.

**Methodology:** Zengfeng Su.

**Writing – review & editing:** Zengfeng Su.

**Writing – original draft:** Tianyun Yu.
